# Single-Cell Transcriptomic and Metabolic Signatures in Exhausted and Classical Memory B Cells—An Exploratory Analysis in Systemic Lupus Erythematosus and Lupus Nephritis

**DOI:** 10.3390/biomedicines14061188

**Published:** 2026-05-25

**Authors:** Litong Zhu, Taoyan Lin, Lai Yee Cheong, Jason K. H. Sher, Irene Y. L. Yam, Wynn Cheung, Susan Yung, Tak Mao Chan, Desmond Y. H. Yap

**Affiliations:** 1 Division of Nephrology, Nanfang Hospital, Southern Medical University, Guangzhou 510515, China; sysuzhu@connect.hku.hk; 2 National Clinical Research Center for Kidney Disease, Nanfang Hospital, Southern Medical University, Guangzhou 510515, China; 3 State Key Laboratory of Multi-Organ Injury Prevention and Treatment, Nanfang Hospital, Southern Medical University, Guangzhou 510515, China; 4 Guangdong Provincial Institute of Nephrology, Nanfang Hospital, Southern Medical University, Guangzhou 510515, China; 5 Guangdong Provincial Key Laboratory of Renal Failure Research, Nanfang Hospital, Southern Medical University, Guangzhou 510515, China; 6 Division of Nephrology, Department of Medicine, School of Clinical Medicine, Queen Mary Hospital, The University of Hong Kong, Hong Kong SAR, China; clyc2204@hku.hk (L.Y.C.); sher2730@hku.hk (J.K.H.S.); iylyam@hku.hk (I.Y.L.Y.); wynncccheung0128@gmail.com (W.C.); ssyyung@hku.hk (S.Y.); dtmchan@hku.hk (T.M.C.); 7 Clinical Pharmacy Center, Nanfang Hospital, Southern Medical University, Guangzhou 510515, China; lint_yan@126.com; 8 Cancer Research Institute, School of Basic Medical Sciences, Southern Medical University, Guangzhou 510515, China

**Keywords:** B cell exhaustion, lupus nephritis, memory B cells, relapse, systemic lupus erythematosus

## Abstract

**Aim:** Disturbances in exhausted and classical memory B cells have been implicated in the pathogenesis of systemic lupus erythematosus (SLE) and lupus nephritis (LN), but the genetic regulation of their homeostasis remains poorly understood. **Methods:** We analyzed the single-cell RNA-seq data of peripheral blood mononuclear cells (PBMCs) from the NIH SLE dataset (GSE135779) and another published LN single-cell RNA-seq dataset (dbGAP database accession code phs001457.v1.p1). Overlapping differentially expressed genes (DEGs) in exhausted and classical memory B cells from SLE and LN patients were identified, and their altered expression was validated in B cells obtained from LN patients. GO and KEGG analyses were used to analyze associated pathways. The relationships between exhausted and classical memory B cells and cellular metabolic pathways were also assessed. **Results:** Three DEGs (IFI44L, XAF1, and MX1) were detected in both exhausted and classical memory B cells, and their increased expression was verified in classical and exhausted memory B cells obtained from LN patients during remission. The protein–protein interaction network of the DEGs suggested that STAT1 showed the highest eigenvector centrality for these DEGs. IFI44L, XAF1 and MX1 were involved in distinct biological processes and immune pathways (especially JAK-STAT). Classical memory B cells showed higher expression of genes involved in sulfur metabolism (SQRDL and TST), amino sugar metabolism (GFPT1 and UAP1), and butanoate metabolism (ACADS and ACAT1), while exhausted B cells exhibited inverse relationships with these metabolic pathways. **Conclusions:** Altered expression of IFI44L, XAF1 and MX1 is associated with distinct metabolic signatures and immune pathways in exhausted and classical memory B cells in SLE and LN.

## 1. Introduction

Systemic lupus erythematosus (SLE) is an autoimmune disorder that affects multiple immunological pathways and organ systems [1]. Lupus nephritis (LN) is a serious complication of SLE, and is associated with various unfavorable short- and long-term clinical outcomes [2]. The advent of immunosuppressive medications used during the induction and maintenance phases has significantly improved outcomes of LN patients over the past two decades [3]. Renal relapses, however, remain a substantial challenge in managing SLE patients, as repeated LN relapses confer increased cumulative treatment-related toxicities and also risk of renal failure [4]. Therefore, novel means that can improve disease monitoring and treatment of SLE and LN patients are eagerly awaited. In this context, a better understanding of the pathogenic mechanisms pertaining to development and relapse in SLE and LN is essential for devising new and effective disease monitoring and therapeutic strategies.

The pathogenesis of SLE and LN involves a complex interplay between genetic predilections, aberrations in innate and adaptive immunity and environmental factors. Abnormalities in the development and function of B lymphocytes occur in different facets of SLE pathogenesis, including defective apoptosis of auto-reactive B cells, expression of aberrant B cell receptors (BCR), presentation of auto-antigens, release of proinflammatory/anti-inflammatory cytokines and synthesis of pathogenic auto-antibodies [5]. Previous studies have reported perturbations of multiple B cell subpopulations in active SLE and LN patients, including a reduction in naive B cells but an increased frequency of classical memory B cells, exhausted B cells and plasma cells in the peripheral blood [6]. Classical memory B cells (CD19^+^CD21^+^CD27^+^) are highly pertinent in the clinical behavior of SLE and LN patients, especially in the propensity for disease relapse. Classical memory B cells possess strong immunological memory and are capable of mounting accentuated immune responses when engaged to previously encountered antigens [7]. Classical memory B cells also show diminished threshold for reactivation and resilience to cell cycle-dependent immunosuppressive agents that are commonly used in the treatment of SLE and LN [8]. B cell exhaustion represents a form of B lymphocyte abnormalities that was first reported in patients with chronic human immunodeficiency virus (HIV) infection. Other investigators had later also observed the phenomenon of B cell exhaustion in certain chronic infections, chronic graft-versus-host disease and autoimmune disorders such as SLE [9]. Exhausted memory B cells (CD19^+^CD21^−^CD27^−^), characterized by downregulation or loss of CD21 along with the upregulation of multiple inhibitory molecules (FCRL4, CD22, CD72, CD85J and CD183), are associated with anomalous BCR signaling, impaired immune response and aberrant expression of chemokines and adhesion molecules [10]. Recent studies have also suggested that alterations in the number and function of classical memory B cells and exhausted B cells were associated with disease relapse in LN patients [5]. Our current knowledge of exhausted B cells remains relatively limited, and the available evidence to date suggests that exhausted B cells may have dual properties. On the one hand, exhausted B cells show impaired calcium mobilization, suppressed proliferation and reduced antibody diversity [11], while on the other hand, they show an increased tendency for auto-reactivity, which may be relevant to the pathogenesis of SLE and LN. Although disturbance in classical and exhausted memory B cells has been noted in SLE and LN patients, the genetic factors that regulate their biology and balance remain poorly understood. Investigating exhausted and classical memory B cells in SLE and LN is highly challenging, as these distinct cell populations are present in very small quantities in circulation. Here, we used the single-cell RNA-seq data of peripheral blood mononuclear cells (PBMCs) from published SLE and LN datasets and performed bioinformatic analyses and relevant in vitro studies to investigate the genetic dysregulation that influences classical and exhausted memory B cells homeostasis in SLE and LN. The results will enhance our knowledge of classical and exhausted memory B cells in SLE and LN, which can potentially be applied in clinical settings to better monitor and control lupus disease activity.

## 2. Patients and Methods

### 2.1. Identifying Differentially Expressed Genes (DEGs)

The single-cell RNA-seq data of PBMCs were obtained from the GSE135779 dataset from the National Institutes of Health (NIH) (dbGaP Study Accession: phs002048.v1.p1), which included 8 adult SLE patients and 6 healthy donors. A separate raw single-cell RNA-seq dataset for LN, comprising data from 24 LN patients, was acquired from a published study (dbGAP database accession code phs001457.v1.p1). To account for potential batch effects between these datasets, raw expression matrices were integrated using Harmony-based batch correction. Principal component analysis (PCA) was performed before and after batch correction to assess its effectiveness (Supplementary Figure S1). Classical memory B cells and exhausted memory B cells were annotated in the scRNA-seq datasets according to canonical marker gene expression profiles. Classical memory B cells were defined by CD19, CD21, and CD27 expression (CD19^+^CD21^+^CD27^+^), whereas exhausted memory B cells were defined by CD19 positivity with reduced or absent CD21 and CD27 expression (CD19^+^CD21^−^CD27^−^). We identified the differentially expressed genes (DEGs) in GSE135779 by comparing the expression profiles between adult SLE patients and healthy controls (HCs) and DEGs by comparing the expression profiles between LN patients (accession code: phs001457.v1.p1) and HCs (accession code: phs002048.v1.p1). The overlapping DEGs between these two datasets were considered potential disease-associated genes, as genes consistently dysregulated across both SLE and LN cohorts were more likely to represent robust and biologically relevant lupus-associated alterations while reducing dataset-specific bias. The differential analysis was performed using the “FindMarkers” function from the Seurat package, and DEGs with adjusted *p* < 0.05 and |log_2_fold change (FC)| > 0.25 were saved. Visualization was conducted using the R package “EnhancedVolcano”. A Venn diagram was created to visualize the overlapping or distinct sets using the R package “ggplot2” (version 3.4.1). A heatmap was then created using “ggplot2” and “reshape2” to display LogFC values for these genes across different conditions.

### 2.2. Validation of Gene Expression by Patient Samples

To validate our bioinformatics findings, we measured the DEGs in classical and exhausted memory B cells obtained from LN patients during disease quiescence (*n* = 10) and from HCs (*n* = 10). Blood samples were obtained from patients with the following inclusion criteria: (1) Patients who have biopsy-proven Class III/IV ± V LN according to the ISN/RPS 2003 classification; (2) Patients in quiescent disease (SLEDAI score < 4 with no points in the renal domain), and on a stable dose of prednisolone of 5–7.5 mg/day (for ≥4 months) alone or in combination with MMF or AZA as maintenance treatment. Patients who received calcineurin inhibitors or proliferation signal inhibitors as maintenance immunosuppression or biologics (e.g., rituximab, abatacept) in the preceding 12 months were excluded. The study protocol was approved by the Institutional Review Board of the University of Hong Kong/Hospital Authority Hong Kong West Cluster (IRB HKU/HKHAWC Reference Number: UW12-447).

Exhausted B cells (CD19^+^CD21^−^CD27^−^) and memory B cells (CD19^+^CD21^+^CD27^+^) were isolated and sorted using the BD Influx™ cell sorter (BD Biosciences, San Jose, CA, USA). Prior to sorting, PBMCs were stained simultaneously with 7-AAD fixable viability dye and fluorophore-conjugated antibodies against CD19, CD21, and CD27 (all from BD Biosciences, Bio-Gene Technology Limited, Hong Kong) to exclude dead cells and label the appropriate cell populations for analysis. The CD19^+^ population was gated first, followed by the gating of the CD21^−^CD27^−^ population as exhausted B cells and the CD21^+^CD27^+^ population as memory B cells. Cell sorting was performed under sterile conditions at 4 °C to preserve cell viability. After sorting, the purity of the populations was validated by running the sorted cells again through the flow cytometer to ensure a purity greater than 90%. Flow cytometry data were analyzed using FlowJo™ software (version 10.8.1). To confirm the purity of the isolated cell populations, flow cytometry plots were generated, showing the gating strategy and the purity of both exhausted and memory B cells post-sorting (Supplementary Figure S2). The purity was consistently above 90%. Following isolation, RNA was extracted from exhausted B cells and memory B cells using ReliaPrep^TM^ RNA Miniprep Systems (Promega, Madison, WI, USA) according to the manufacturer’s instructions. mRNA was converted to cDNA, and the levels were quantified by qPCR. The PCR primers (Supplementary Table S1) were synthesized by Integrated DNA Technologies (Coralville, Johnson County, IA, USA).

### 2.3. Gene Expression Correlation Heatmap

A gene expression correlation heatmap was performed by the “corrplot” package (version 0.92). This entailed the computation of correlation coefficients, assessment of *p*-values, and the subsequent visualization of the data. The analysis utilized an scRNA-seq public dataset consisting of 8 adult SLE patients and 6 HCs. Scatter plots for gene pairs (XAF1, STAT1, IFI44L, MX1) were created using the rcorr function from the Hmisc package to visualize Pearson correlations and *p*-values.

### 2.4. Protein–Protein Interaction (PPI) Network Analysis

We used the Search Tool for Retrieval of Interacting Genes (STRING) database (https://string-db.org, accessed on 4 May 2026), which integrates both known and predicted PPIs, to analyze the functional interactions of proteins related to DEGs identified. Furthermore, to ensure the validity and pertinence of our results, we considered only those interactions with a combined score > 0.4 as statistically significant. Cytoscape version 3.6.1 was used to visualize the PPI network. Comprehensive analysis of the eigenvector centrality was performed based on the values of Degree Centrality, Betweenness Centrality, and Closeness Centrality.

### 2.5. Western Blot Analysis of STAT1 in Exhausted B Cells

Exhausted B cells from LN patients and healthy controls were lysed in RIPA buffer containing protease and phosphatase inhibitors, and protein concentrations were determined using the BCA assay. Equal amounts of protein (20–30 μg) were separated by SDS-PAGE and transferred onto PVDF membranes, which were blocked with 5% non-fat milk in TBST for 1 h at room temperature. Membranes were then incubated overnight at 4 °C with STAT1 monoclonal antibody (Proteintech, Cat. No. 66545-1-Ig, 1:5000) and α-Tubulin mAb (Abclonal, Cat. No. A6830, 1:10,000) as loading control. After washing, membranes were incubated with HRP-conjugated secondary antibodies for 1 h at room temperature. Bands were visualized using enhanced chemiluminescence (ECL) and quantified with ImageJ software (version 1.54d; National Institutes of Health, Bethesda, MD, USA), and STAT1 protein levels were normalized to α-tubulin.

### 2.6. Gene Ontology (GO) and Pathway Enrichment Analysis of DEGs

Enrichment analysis and immune landscape analysis were performed by R Studio (R version 4.1.0). Both Gene Ontology (GO) enrichment analysis and Kyoto Encyclopedia of Genes and Genomes (KEGG) pathway enrichment analyses based on the identified DEGs were performed using the “clusterProfiler” package (version 4.7.1003). The analysis was based on overlapping DEGs obtained from the comparison between SLE vs. HC and LN vs. HC, followed by PPI network analysis to identify a set of 69 key genes. The functional enrichment result was shown using the “ggplot2” package (version 3.4.1).

### 2.7. Cell Metabolic Analysis

We also examined the metabolic pathways related to the identified DEGs, as mounting evidence has suggested that disturbance in cell metabolism may affect immune cell functions [12]. The metabolic pathway analysis was based on DEGs from an scRNA-seq public dataset comprising 8 adult SLE patients and 6 HCs. The “scMetabolism” packages (version 0.2.1) were used to quantify single-cell metabolic activity (PMID: 34417225), and the results were visualized using the “pheatmap” package (version 3.4.1).

### 2.8. Validation of Key Genes in Metabolic Pathways

We validated the differential expression of six key genes involved in sulfur metabolism (SQRDL, TST), amino sugar and nucleotide sugar metabolism (GFPT1, UAP1), and butanoate metabolism (ACADS, ACAT1) using the “Seurat” package. The data used for validation were derived from a scRNA-seq public dataset, which included 8 adult SLE patients and 6 HCs. Differential expression analysis between memory B cells and exhausted B cells was performed, and results were visualized as bar plots with standard error bars using the “ggplot2” package.

### 2.9. Statistical Analysis

Continuous variables were analyzed by Student’s *t*-test or the Mann–Whitney U-test as appropriate. Correlation analysis between genes was performed using the Spearman correlation coefficient. All statistical analyses were performed using the R software (version 4.1.0) and GraphPad Prism 9.5.1 (GraphPad Software, San Diego, CA, USA). Results were considered statistically significant when the two-tailed *p*-value was < 0.05.

## 3. Results

### 3.1. IFI44L, XAF1, and MX1 Are Consistently Upregulated in Classical Memory and Exhausted B Cells from SLE and LN Patients

**Figure 1:**
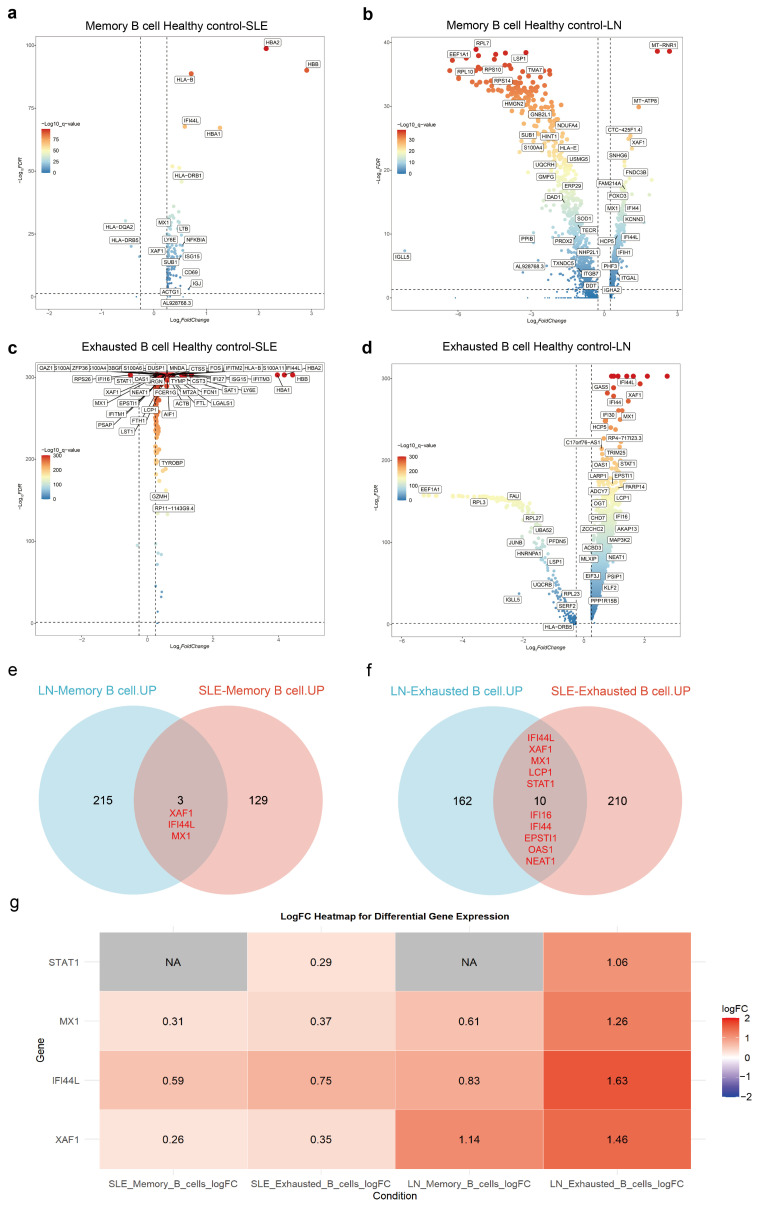
Identification and comparison of differentially expressed genes (DEGs) in B cell subsets across SLE and LN cohorts. (**a**–**d**) Volcano plots showing DEGs in (**a**,**b**) memory B cells and (**c**,**d**) exhausted B cells from patients with SLE and LN compared to healthy controls. Each dot represents a gene, with upregulated genes located on the right side. (**e**,**f**) Venn diagrams showing overlapping DEGs in (**e**) classical memory B cells and (**f**) exhausted B cells between SLE and LN groups. IFI44L, XAF1, and MX1 are identified as overlapping genes in both cell types, showing higher expression in LN. (**g**) Heatmap of logFC values illustrates the expression changes of these overlapping genes, distinguishing between shared and cell-type-specific movements. The thresholds for DEGs were defined as an adjusted *p*-value < 0.05 and |log_2_FC| > 0.25.

We first investigated the DEGs in classical memory B cells between SLE patients, LN patients and HCs (Figure 1a,b). Next, we also studied the DEGs in exhausted B cells between SLE patients, LN patients and HCs (Figure 1c,d). IFI44L, XAF1 and MX1 were identified as the overlapping DEGs in memory B cells between LN and SLE patients, showing higher expression in LN patients (Figure 1e), while IFI44L, XAF1, MX1, LCP1, STAT1, IFI16, IFI44, EPSTI1, OAS1, and NEAT1 were the overlapping DEGs in exhausted B cells between SLE and LN patients, also showing higher expression in LN patients (Figure 1f). Here, we found that IFI44L, XAF1 and MX1 were DEGs upregulated in both exhausted and classical memory B cells. Our LogFC heatmap further demonstrated consistent upregulation in both exhausted and memory B cells, with STAT1 showing cell-type-specific expression in exhausted B cells (Figure 1g). These results suggest that IFI44L, XAF1, and MX1 may play significant roles in the immune dysfunction seen in SLE and LN, particularly in LN patients, where their expression is elevated in both classical memory and exhausted B cells.

### 3.2. IFI44L, XAF1, and MX1 Are Consistently Upregulated in Classical Memory and Exhausted Memory B Cells from Patients with LN

**Figure 2:**
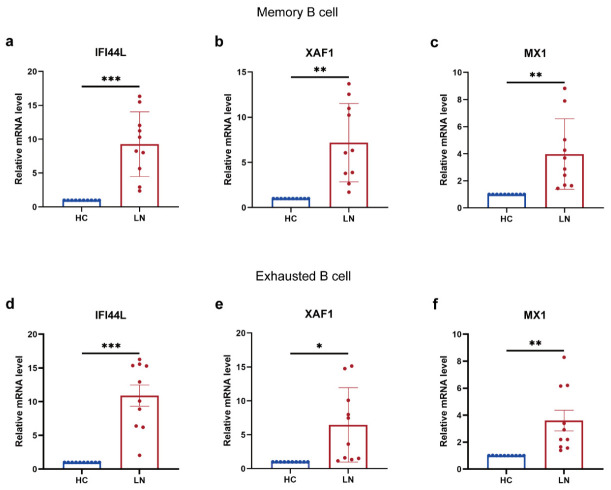
The expression of IFI44L, XAF1 and MX1 in classical memory and exhausted B cells from lupus nephritis (LN) patients and healthy controls (HCs). The expression levels of (**a**) IFI44L, (**b**) XAF1 and (**c**) MX1 in classical memory B cells were significantly higher in the LN group compared to the HC group. Exhausted B cells from LN patients also showed higher expression of (**d**) IFI44L, (**e**) XAF1 and (**f**) MX1 than those from HCs. * *p* < 0.05, ** *p* < 0.01, *** *p* < 0.001.

To validate our bioinformatics analyses, we compared the mRNA levels of IFI44L, XAF1, and MX1 in classical memory and exhausted B cells obtained from LN patients during disease remission and from HCs. The expressions of IFI44L, XAF1, and MX1 in classical memory B cells from LN patients were significantly higher than those from HCs (Figure 2a–c). LN patients also showed significantly increased levels of IFI44L, XAF1, and MX1 in exhausted B cells compared to HCs (Figure 2d–f). These results validated our bioinformatics predictions, confirming that IFI44L, XAF1, and MX1 are significantly upregulated in both classical memory and exhausted B cells in LN patients compared to healthy controls, supporting their potential role in LN pathogenesis.

### 3.3. Integrated Correlation and Protein–Protein Interaction (PPI) Network Analysis of Key Candidate Genes

**Figure 3:**
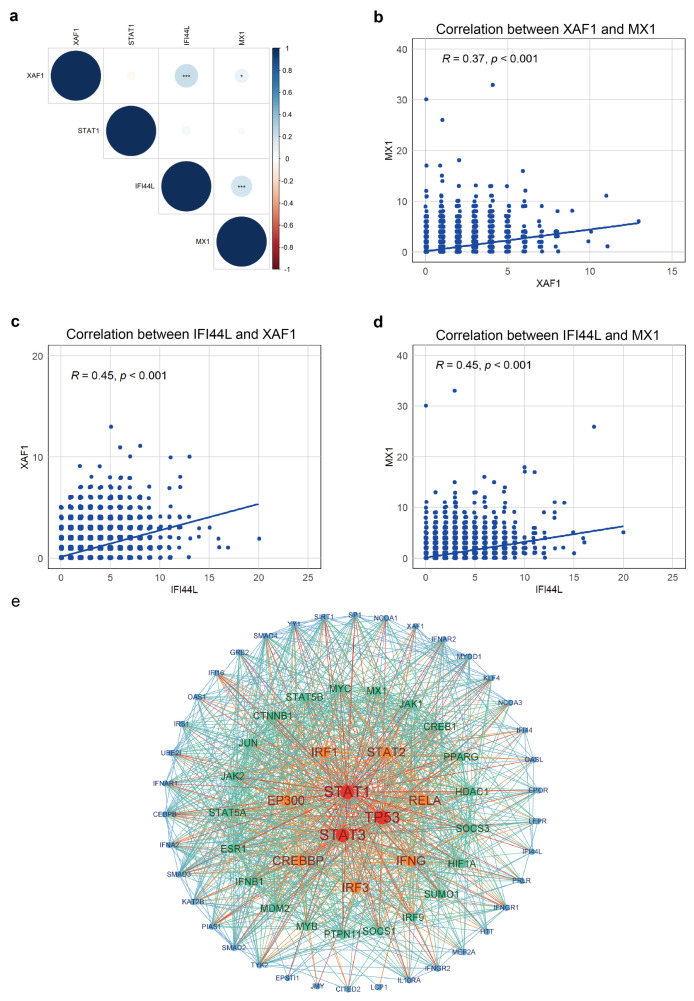
Correlation and protein–protein interaction (PPI) analysis of key differentially expressed genes. (**a**) XAF1 exhibited a mild positive correlation with MX1 in SLE patients (r = 0.08, *p* < 0.05). Furthermore, IFI44L demonstrated highly significant positive correlations with both XAF1 and MX1 in SLE patients (r = 0.21, 0.18; both *p* < 0.001). Scatter plots were generated to further explore these relationships. (**b**) The plot for XAF1 and MX1 shows a positive correlation (r = 0.37, *p* < 0.001). (**c**) The plot for IFI44L and XAF1 reveals a moderate positive correlation (r = 0.45, *p* < 0.001). (**d**) Similarly, a positive correlation was observed between IFI44L and MX1 (r = 0.45, *p* < 0.001). (**e**) Based on the combined DEG set from both classical and exhausted memory B cell subsets, STAT1, STAT2, and TP53 showed good eigenvector centrality based on the evaluation of Degree Centrality, Betweenness Centrality, and Closeness Centrality. * *p* < 0.05, *** *p* < 0.001.

The relationships between IFI44L, XAF1, MX1 and STAT1 were assessed by the gene expression correlation heatmap. XAF1 showed a slight positive correlation with MX1 in SLE patients (r = 0.08, *p* < 0.05). Moreover, IFI44L exhibited highly significant positive correlations with both XAF1 and MX1 in SLE patients (r = 0.21, 0.18, all *p* < 0.001) (Figure 3a). In addition, scatter plots were generated to further illustrate the relationships between these genes. The scatter plot shows a positive correlation between XAF1 and MX1 (r = 0.37, *p* < 0.001) (Figure 3b). A moderate positive correlation was observed between IFI44L and XAF1 (r = 0.45, *p* < 0.001) (Figure 3c), and a similar correlation was found between IFI44L and MX1 (r = 0.45, *p* < 0.001) (Figure 3d). These correlation analyses confirmed the significant positive correlations between IFI44L, XAF1, and MX1, with weaker but still notable correlations between XAF1 and MX1. The PPI network of DEGs, which was based on the STRING database, included 69 DEGs. The proteins IRF1, IRF3, EP300, CREBBP, STAT1, STAT2, STAT3, RELA, IFNG, and TP53 showed good eigenvector centrality based on the evaluation of Degree Centrality, Betweenness Centrality, and Closeness Centrality (Figure 3e). Among these proteins, STAT1 appeared to show the highest eigenvector centrality. The elevated STAT1 expression in exhausted B cells from LN patients was confirmed by both Western blot and qPCR (Supplementary Figure S3). The PPI network analysis identified STAT1 as a hub protein of potential relevance, and its increased expression in exhausted B cells suggests a possible association with the molecular alterations observed in LN.

### 3.4. Functional Enrichment Analysis Reveals Significant Involvement of DEGs in Immune-Inflammatory Responses and Cellular Stress Management

**Figure 4:**
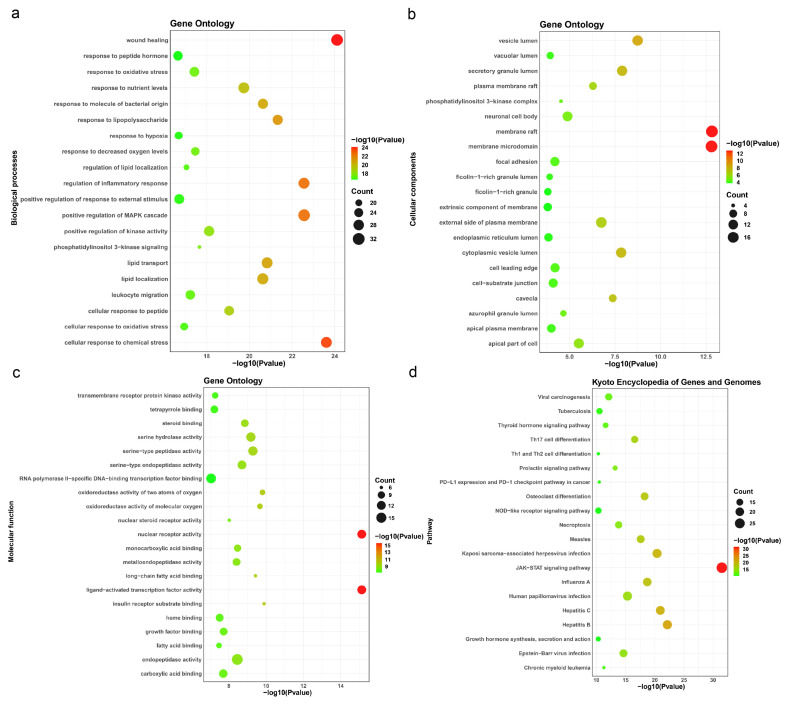
Functional enrichment and pathway analysis of differentially expressed genes. (**a**–**c**) Bubble charts showing the top 20 most significantly enriched Gene Ontology (GO) terms in (**a**) biological processes (BP), (**b**) cellular components (CCs), and (**c**) molecular functions (MFs). The vertical axis lists the specific GO terms for each category, while the horizontal axis displays the corresponding −log_10_(*p*-value), indicating the significance of enrichment. (**d**) Bubble chart showing the top 20 most significantly enriched pathways from the Kyoto Encyclopedia of Genes and Genomes (KEGG) analysis, with the vertical axis listing the KEGG pathways and the horizontal axis representing the −log_10_(*p*-value).

GO and KEGG enrichment analyses were performed to investigate the functions and pathways of DEGs, respectively. For biological processes (BPs), gene expressions were significantly enriched in processes related to wound healing, positive regulation of the MAPK cascade, regulation of inflammatory response, lipid localization, cellular response to chemical stress, and response to nutrient levels (Figure 4a). For the cellular component (CC) analysis, gene expressions were significantly enriched in the membrane raft, membrane microdomain, vesicle lumen, secretory granule lumen, and external side of the plasma membrane (Figure 4b). As for molecular function (MF), gene expressions were significantly enriched in serine-type peptidase activity, serine hydrolase activity, serine-type endopeptidase activity, nuclear receptor activity, ligand-activated transcription factor activity, and endopeptidase activity (Figure 4c). The KEGG pathway enrichment analysis further showed significant involvement in pathways related to JAK-STAT signaling and viral infections (including human papillomavirus infection, hepatitis B, hepatitis C, Kaposi sarcoma-associated herpesvirus infection, and Epstein-Barr virus infection) (Figure 4d). Our GO and KEGG enrichment analyses highlighted that DEGs are primarily involved in immune and inflammatory responses, cellular stress management, and key signaling pathways like JAK-STAT, suggesting their potential role in regulating immune dysfunction and virus-related mechanisms.

### 3.5. Characterization of Metabolic Pathways and Key Gene Expression Profiles in Classical Memory and Exhausted B Cells from Patients with SLE and LN

**Figure 5:**
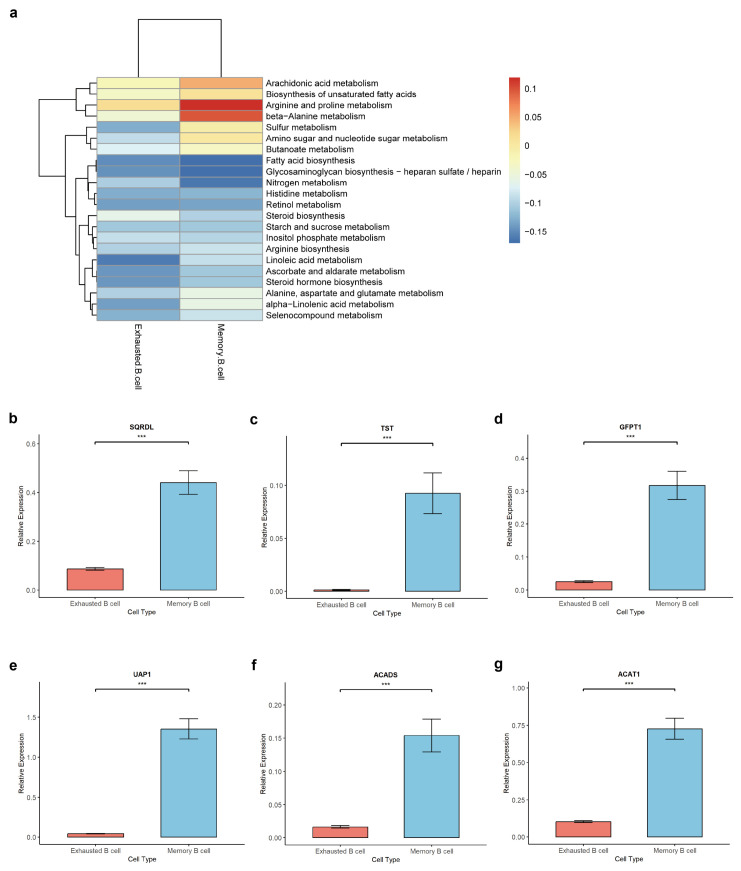
Divergent metabolic landscapes and key gene expression profiles in classical memory versus exhausted B cells from patients with SLE and LN. (**a**) Overview of the relationship between B cell subsets and metabolic pathways. Classical memory B cells exhibit positive correlations with sulfur metabolism, amino sugar and nucleotide sugar metabolism, and butanoate metabolism, whereas exhausted B cells demonstrate inverse relationships with these pathways. (**b**–**g**) Relative expression levels of key metabolic genes in memory and exhausted B cells. SQRDL (**b**) and TST (**c**), which are involved in sulfur metabolism, were significantly upregulated in memory B cells compared to exhausted B cells. Similarly, GFPT1 (**d**) and UAP1 (**e**), associated with amino sugar and nucleotide sugar metabolism, showed higher expression in memory B cells. Finally, genes related to butanoate metabolism, ACADS (**f**) and ACAT1 (**g**), also demonstrated significantly higher expression levels in memory B cells. *** *p* < 0.001.

Functional enrichment analysis revealed distinct metabolic profiles between B cell subsets. Classical memory B cells exhibited significant positive correlations with sulfur metabolism, amino sugar and nucleotide sugar metabolism, and butanoate metabolism, whereas exhausted B cells showed inverse relationships with these pathways (Figure 5a). These findings suggest that classical memory B cells maintain robust metabolic activity to support energy production and stress management, whereas the reduced metabolic signature in exhausted B cells may contribute to their impaired effector functions in SLE and LN. To further validate these metabolic shifts, we examined the expression of six representative genes. In the sulfur metabolism pathway, ***SQRDL*** and ***TST*** were significantly upregulated in classical memory B cells compared to exhausted B cells (Figure 5b,c). Similarly, regarding amino sugar and nucleotide sugar metabolism, both ***GFPT1*** and ***UAP1*** showed higher expression in the classical memory subset (Figure 5d,e). Furthermore, genes involved in butanoate metabolism, specifically ***ACADS*** and ***ACAT1***, were also significantly more abundant in classical memory B cells than in exhausted B cells (Figure 5f,g). Collectively, these results indicate that classical memory B cells possess an enhanced metabolic capacity across these specific pathways, underscoring the functional divergence between memory and exhausted B cell populations in the context of lupus pathogenesis.

## 4. Discussion

Perturbations in exhausted and classical memory B cells are observed in SLE and LN patients [13], but the mechanisms that orchestrate their balance and biology remain elusive. Our results suggested that distinct DEGs were related to the homeostasis of exhausted and classical memory B cells in SLE and LN. Our data also indicated that these DEGs were involved in various important cellular/molecular functions of immune cells in SLE and LN.

The results from our bioinformatics analyses showed that dysregulation of IFI44L, XAF1 and MX1 was present in both classical and exhausted memory B cells in LN patients. We further validated the increased expression of IFI44L, XAF1 and MX1 in classical and exhausted memory B cells obtained from LN patients during disease remission, which corroborated our findings by bioinformatics analyses. IFI44L (interferon-inducible 44-like) is a newly discovered interferon-inducible gene, which was originally identified in the study of immune responses to viruses. Overexpression of IFI44L is associated with various autoimmune diseases, including rheumatoid arthritis (RA), systemic sclerosis (SSc), Sjogren’s syndrome and SLE. Recent studies have also suggested that the methylation status of the IFI44L gene’s promoter region might serve as a biomarker for the early detection of SLE [14]. XAF1 (X-linked inhibitor of apoptosis-associated factor 1) is a type 1 interferon (IFN)-inducible gene that encodes a zinc finger protein with pro-apoptotic properties. XAF1 has been largely studied in carcinogenesis and is generally regarded as a tumor-suppressor gene, as its expression is often downregulated or silenced in various types of cancer. XAF1 negatively regulates type 1 IFN production in viral infections, but our understanding of the role of XAF1 in autoimmunity, including LN, is still relatively limited [15]. MX1, also known as MX Dynamin-like GTPase 1, plays a crucial role as an antiviral gene in humans. The transcription of MX1 is stimulated by IFN-β, which is produced in response to TLR3 or TLR4 activation [16]. Beyond its upregulation in LN, increased levels of MX1 have also been observed in other autoimmune conditions, such as RA, idiopathic interstitial pneumonia, and alopecia areata [17].

Type 1 IFN signaling plays a pivotal role in the pathogenesis of SLE, with a significant proportion of patients exhibiting elevated Type 1 IFN signatures. This increased Type 1 IFN activity is typically induced by toll-like receptor (TLR) signaling, particularly TLR7/8/9, as a response to viral infections and nucleic acid-containing immune complexes. In this study, the identified DEGs (IFI44L, XAF1, MX1) are recognized as interferon-stimulated genes (ISGs). Together with LN-expressed genes such as OAS1, IFI44, and IFI16, as pointed out by the reviewer, all cumulatively support an enhanced type 1 IFN response in SLE and LN, indicating that enhanced Type 1 IFN signaling may underlie the observed gene expression patterns in LN. We acknowledge that IFI44L, XAF1, MX1, and STAT1 are classical interferon-related genes and may also be dysregulated in other immune cell populations under inflammatory conditions. Therefore, these findings should be interpreted as B-cell-subset-associated changes within an IFN-rich immune context, rather than definitive evidence of absolute cellular specificity.

Although our results showed correlations between IFI44L, XAF1 and MX1 expression in SLE patients, the exact interaction between these DEGs and their roles in SLE pathogenesis remains to be investigated. In our protein–protein interaction analysis, STAT1 appeared to show the highest eigenvector centrality and, hence, might be a common pathway involved by IFI44L, XAF1 and MX1. Our KEGG pathway enrichment analysis, which showed significant involvement of JAK-STAT signaling, further supported the potential involvement of STAT1 in SLE and LN. STAT1 (Signal Transducer and Activator of Transcription 1) is one of the transcription factors in the STAT family that plays a crucial role in regulating immune responses and the IFN signaling pathway. STAT1 also modulates various cellular processes, including cell proliferation, apoptosis and differentiation, within the context of metabolism. Overactive STAT1 may enhance the production of various inflammatory mediators and cytokines, including tumor necrosis factor-alpha (TNF-α) and interleukin-6 (IL-6), thereby causing renal parenchymal damage in LN [18]. Altered STAT1 activity has also been implicated in the pathogenesis of inflammatory bowel disease, viral replication, immunodeficiency syndromes and various types of cancer [19]. Our GO and KEGG pathway analyses also suggested that IFI44L, XAF1 and MX1 were involved in biological processes and pathways related to wound healing, MAPK cascade, regulation of inflammatory response, lipid localization, cellular response to chemical stress and nutrient levels, and various enzymatic activities, all of which may contribute to the pathogenesis of SLE and LN. Impaired damage healing in SLE results from chronic inflammation and immune dysregulation, contributing to persistent tissue injury in LN [20]. The MAPK signaling cascade regulates immune responses, and its aberrant activation is linked to chronic inflammation and tissue injury [21]. Dysregulated inflammatory pathways further exacerbate inflammation, leading to immune-mediated kidney damage [22]. Lipid localization affects immune cell membrane function and B cell signaling [23], while chronic cellular stress responses in SLE may overwhelm the immune cells’ capacity to cope with oxidative stress, impairing their function [24]. Disruptions in nutrient regulation affect immune metabolism and chronic activation in SLE, and altered enzymatic activities contribute to metabolic dysfunctions in lupus nephritis [25]. These alterations constitute the complex interplay between immune responses, metabolism, and cellular stress in SLE and LN. Our results also indicated that classical and exhausted memory B cells showed significant correlations with distinct metabolic pathways. Of note, exhausted B cells showed negative associations with various metabolic pathways, which was in line with previous studies that reported reduced metabolic activities in exhausted B cells [26]. Emerging evidence also suggests that disturbance in the metabolic environment may affect T lymphocyte functions in autoimmunity, including SLE [12]. However, the associations between metabolic pathways and B cell subsets are largely correlative, and it is possible that the observed metabolic changes are secondary consequences rather than primary drivers of B cell subset dysfunction. One might also postulate that our findings were related to broader inflammatory activation and differences in B-cell activation/differentiation states. The metabolic regulation of the B-cell repertoire in autoimmune conditions remains poorly understood but is a worthwhile area to be further studied. Our transcriptomic data revealed a broad downregulation of key metabolic networks—including sulfur, amino sugar, and butanoate metabolism—in exhausted B cells compared to classical memory B cells. While canonical pathways like glycolysis and glutaminolysis are fundamental for general B-cell activation, our subset-specific comparison highlighted these alternative pathways as key differentiators. Rather than merely reflecting cellular quiescence, this metabolic insufficiency likely underpins their impaired regulatory capacity and characteristic state of exhaustion in SLE. For instance, diminished sulfur metabolism compromises cellular redox balance and the ability to withstand oxidative stress [27], while reduced amino sugar pathways may impair glycosylation processes essential for proper protein folding and receptor signaling [28]. Conversely, the metabolic flexibility retained by classical memory B cells—such as utilizing butanoate to fuel the mitochondrial TCA cycle—enables them to meet immense energetic demands alongside canonical pathways [29]. This distinct metabolic reprogramming between B cell subsets strongly aligns with emerging evidence that metabolic disturbances govern immune cell fate and dysfunction in autoimmune environments [12,26]. Further studies are needed to clarify the exact role these metabolic pathways play in B-cell function and how they could be targeted for therapeutic interventions in SLE and LN. The exact role of exhausted B cells in the pathogenesis of SLE and LN remains elusive. While impaired humoral immunity has been reported in B cell exhaustion, some studies suggested that exhausted B cells showed increased auto-reactive tendency [30]. Such observations underscore the potential pathogenic relevance of exhausted B cells in the development and progression of SLE and LN.

One important limitation of this study was that the data was largely derived from bioinformatics analysis, and hence, the findings were mainly associative and the pathogenic roles of the identified DEGs could not be confidently established. The expression of DEGs in classical and exhausted memory B cells was only measured in LN during remission but not compared with that during active nephritis, and hence, the expressions of IFI44L, XAF1 and MX1 in active LN were unknown. Also, additional cell surface markers such as T-bet, CD11c, and FCRL4 were not included in our sorting panel. Consequently, the CD19^+^CD21^−^CD27^−^ “exhausted” B cell population isolated in our validation cohort is heterogeneous and remains possible to overlap with age-associated B cells (ABCs) or atypical memory B cells. Because ABCs and atypical memory B cells possess distinct functional properties, such as altered activation thresholds and the capacity to produce autoantibodies, the transcriptomic and protein expression changes observed in our study (such as STAT1, IFI44L, XAF1, and MX1) might partially reflect the characteristics of these overlapping B cell subsets. Future studies employing higher-dimensional flow cytometry or single-cell approaches are required to precisely delineate these specific B cell subsets in SLE and LN. Notwithstanding, to enhance the reliability of our results, we measured the transcript levels of the identified DEGs in classical and exhausted memory B cells obtained from LN patients in our centre. Furthermore, results were not validated in kidney biopsy samples collected from LN patients. Here, we only used data from public domains for bioinformatics analysis, and hence, the single-cell data of some uncommon cell types (e.g., exhausted B cells) within the kidneys may not be fully available. Another limitation was that the data from the public domain did not include important clinical information, such as lupus manifestations, serological parameters and SLEDAI, to correlate the findings with disease activity states. Nonetheless, our data might provide a scientific basis to embark on translational/animal studies to investigate the role of IFI44L, XAF1 and MX1 in LN and their clinical applications as biomarkers or therapeutic targets. The results of these future studies may help improve our disease monitoring and treatment strategies for SLE and LN patients. Another caveat in interpreting our results was that the LN and SLE/HC cohorts were sequenced separately, where sequencing-platform-derived differences may have influenced the results. To minimize platform differences, we applied standard batch correction techniques commonly used in transcriptomic studies, ensuring that the downstream analysis focused on biologically relevant variations rather than technical artifacts. Ideally, one can also examine non-immune relevant genes to evaluate platform effects. Nonetheless, the consistency of immune-related gene expression patterns observed across the LN and SLE/HC cohorts suggests that any residual platform differences are unlikely to significantly impact our main findings. Furthermore, the immune-related DEGs identified are consistent with known lupus pathophysiology, lending further support for the biological relevance of our results. Future studies are needed to explore the functional roles of IFI44L, XAF1, and MX1 in B cell regulation and their involvement in the JAK-STAT signaling pathway. Additionally, investigating the metabolic pathways identified in our analysis could provide new perspectives in restoring immune balance in SLE and LN. Analyzing DEGs between SLE and LN patients may also help identify unique nephritis-associated signatures distinct from background autoimmunity.

Despite these limitations, this study provides novel insights into the genetic regulation of exhausted and classical memory B cells in SLE and LN. The identification of key dysregulated genes such as IFI44L, XAF1, and MX1, and their involvement in the JAK-STAT signaling pathway, highlights some critical mechanisms for driving immune dysfunction in SLE and LN. In addition, we found significant differences in the expression of metabolic pathway genes, including those involved in sulfur, amino sugar and butanoate metabolism, with classical memory B cells showing upregulation of these pathways compared to exhausted B cells. This dual dysregulation of both immune and metabolic pathways provides a more comprehensive understanding of the distinct roles these B cell subtypes play in SLE and LN. Our findings are clinically relevant, as these DEGs hold promise for the development of biomarkers to track disease activity, predict relapses, and monitor therapeutic responses. For instance, IFI44L could be utilized as a biomarker in early-stage disease detection and prediction of relapse in LN patients [14]. Moreover, the identification of JAK-STAT signaling and metabolic gene dysregulation as potential therapeutic targets opens the avenue for restoring B cell homeostasis in SLE and LN via attenuation of these pathways (e.g., JAK inhibitors).

## 5. Conclusions

Altered IFI44L, XAF1 and MX1 expression in classical and exhausted memory B cells is associated with altered metabolic signatures in SLE and LN. Dysregulated IFI44L, XAF1 and MX1 expression correlates with distinct immune pathways and biological processes pertinent to SLE and LN, although a causal role in disease pathogenesis remains to be established.

## Data Availability

The data generated or analyzed during this study, along with relevant materials, are included in this manuscript and its Supplementary Files.

## Supplementary Materials

The following supporting information can be downloaded at: https://www.mdpi.com/article/10.3390/biomedicines14061188/s1, Figure S1: PCA plots of single-cell RNA-seq data from systemic lupus erythematosus (SLE), lupus nephritis (LN), and healthy donor cohorts before and after Harmony-based batch correction; Figure S2: Gating strategy and purity of classical memory B cells and exhausted B cells; Figure S3: The expression of STAT1 in exhausted B cells from LN patients and HCs. STAT1 expression was examined in exhausted B cells isolated from LN patients and HCs; Table S1: Primers for real time-quantitative PCR.
