# Supplementary material for: Single-Cell Transcriptomic and Metabolic Signatures in Exhausted and Classical Memory B Cells—An Exploratory Analysis in Systemic Lupus Erythematosus and Lupus Nephritis
Source: Biomedicines. 2026 May 25;14(6):1188. doi: 10.3390/biomedicines14061188 (PMC13295815; doi:10.3390/biomedicines14061188)

Supplementary Table S1: Primers for real time-quantitative PCR.

Gene	Direction	Sequence (5’ to 3’)
STAT1	Forward	5'-ATGGCAGTCTGGCGGCTGAATT-3'
	Reverse	5'-CCAAACCAGGCTGGCACAATTG-3'
IFI44L	Forward	5'-TGCACTGAGGCAGATGCTGCG-3'
	Reverse	5'-TCATTGCGGCACACCAGTACAG-3'
XAF1	Forward	5'-GTGTCCTGCTTGGTGCCTGAATC-3'
	Reverse	5'-GTCCTTCCGTCCCTTTCTACAGTTC-3'
MX1	Forward	5'-GGCTGTTTACCAGACTCCGACA-3'
	Reverse	5'-CACAAAGCCTGGCAGCTCTCTA-3'
18sRNA	Forward	5'-CTACCACATCCAAGGAAGCA-3'
	Reverse	5'- TTTTTCGTCACTACCTCCCCG-3'

Supplementary Figure S1. PCA plots of single-cell RNA-seq data from SLE, LN, and healthy donor (HD) cohorts before and after batch correction. (**a, b**) SLE vs Healthy control: The left panel (a) shows PCA prior to batch correction, where SLE and healthy samples exhibit cohort-specific clustering, reflecting technical differences. The right panel (b) shows PCA after Harmony-based batch correction, demonstrating improved alignment between the SLE and healthy samples while preserving biological variability. (**c, d**) LN vs Healthy control: The left panel (c) shows PCA before batch correction, with clear separation of LN and healthy samples due to batch effects. The right panel (d) shows PCA after Harmony correction, indicating effective mitigation of batch effects and better integration of the cohorts while maintaining biological differences.

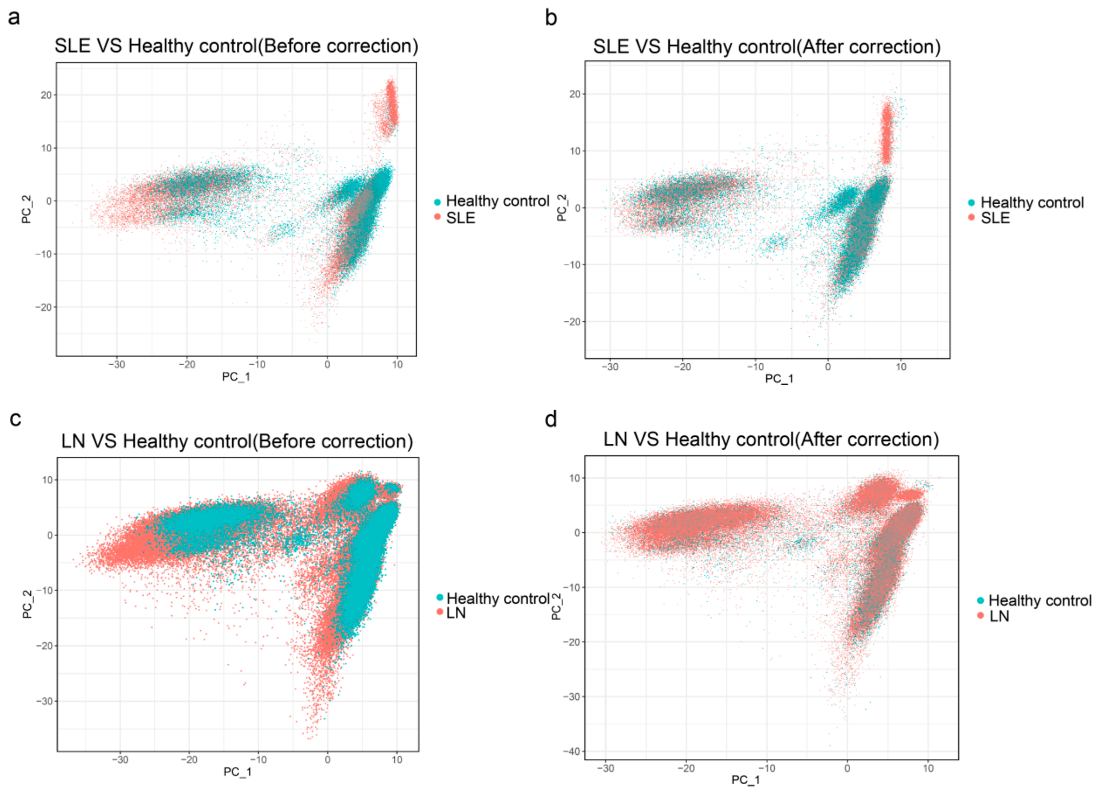

Supplementary Figure S2. Gating strategy and purity of classical memory B cells and exhausted B cells. Flow cytometry was used to isolate classical memory B cells (CD19+CD21+CD27+) and exhausted B cells (CD19+CD21-CD27-). The gating strategy, shown in panels **a-d**, involved size and granularity selection (FSC vs SSC), exclusion of doublets (Trigger Pulse Width), and dead cells using 7-AAD. Live CD19+ B cells were gated, and subpopulations were identified by CD21 and CD27 expression. Post-sorting, the purity of both populations was confirmed in panels **e** and **f**, with purities consistently above 90%, ensuring reliable downstream analyses.

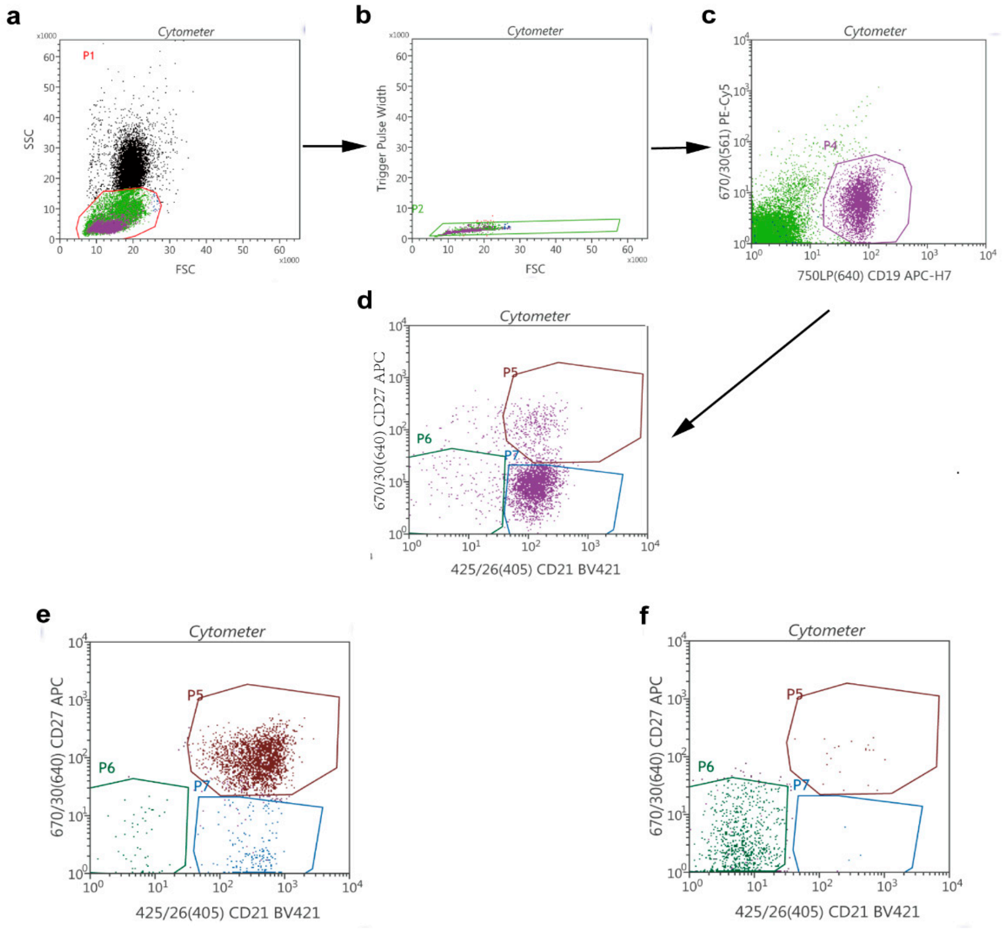

Supplementary Figure S3. The expression of STAT1 in exhausted B cells from lupus nephritis (LN) patients and healthy controls (HC). **(a)** Representative Western blot showing STAT1 protein levels in exhausted B cells from HC and LN patients; α-tubulin was used as loading control. **(b)** Quantification of STAT1 protein levels normalized to α-tubulin. LN patients exhibited significantly higher STAT1 expression than HC. **(c)** Relative STAT1 mRNA levels measured by qPCR, showing elevated expression in LN compared to HC. Data are presented as mean ± SEM; statistical significance: ***$p < 0.001$, ****$p < 0.0001$.

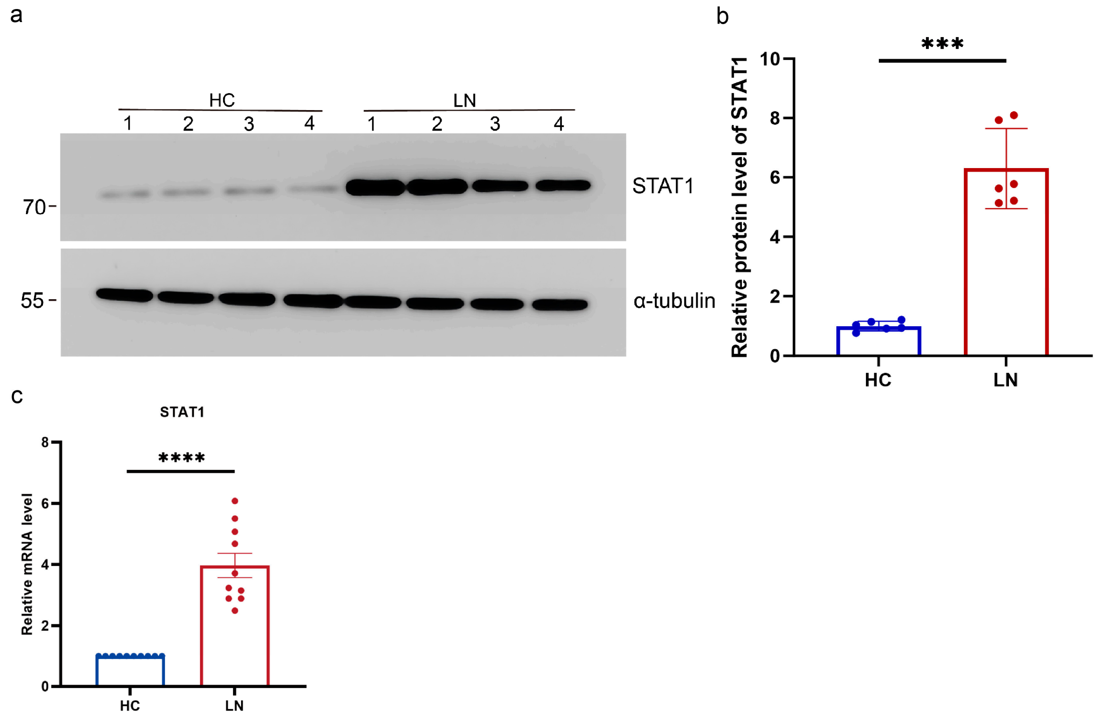

Supplement: Supplementary file 1 [file biomedicines-14-01188-s001.zip › biomedicines-4230270-supplementary.pdf]
